# Targeted therapy with vemurafenib in BRAF(V600E)-mutated anaplastic thyroid cancer

**DOI:** 10.1186/s13044-023-00147-7

**Published:** 2023-03-01

**Authors:** Matthias Lang, Thomas Longerich, Chrysanthi Anamaterou

**Affiliations:** 1 Department of General-, Visceral- and Transplant Surgery, University Hospital Heidelberg, Heidelberg, Germany; 2 Institute of Pathology, University Hospital Heidelberg, Heidelberg, Germany; 3 Department of Nuclear Medicine, University of Heidelberg, Heidelberg, Germany

**Keywords:** Anaplastic thyroid carcinoma, Vemurafenib, BRAF-inhibitor, Adjuvant targeted therapy, BRAF(V600E)-mutation, Review

## Abstract

**Background:**

Anaplastic thyroid cancer (ATC) is one of the most aggressive malignancies, representing less than 5% of all thyroid carcinomas. Τhe median survival is limited to months due to the resistance of ATC to surgery, radioiodine therapy, radiotherapy and chemotherapy. This review will cover novel agents involving several cellular signaling pathways including the BRAF pathway. The BRAF inhibitor vemurafenib improves survival among patients with metastatic melanoma, hairy-cell leukemia and intracranial neoplasms with BRAF gene mutations. The frequency of a BRAF (V600E) mutation in ATC is about 25%.

**Case presentation:**

We report the first case of a marked partial response to adjuvant first line monotherapy with vemurafenib in BRAF V600E-mutated ATC. The 78-year-old man showed a sustained response for 7 months, thereafter scans revealed progressive disease and the patient died 10 months after first diagnosis. This case report is accompanied by a comprehensive review of current strategies and tools for ATC treatment.

**Conclusions:**

This case and the review of current data confirm the benefit of BRAF inhibition in BRAF-mutated ATC, limited by acquired resistance to targeted therapy.

**Supplementary Information:**

The online version contains supplementary material available at 10.1186/s13044-023-00147-7.

## Background

Anaplastic thyroid cancer (ATC) is a highly virulent malignant condition. Although it represents only 2–5% of all thyroid tumors, it is responsible for up to 40% of thyroid carcinoma-related deaths [1, 2]. ATC cells do not retain any of the biological features of the original follicular cells, such as uptake of iodine and synthesis of thyroglobulin, contributing to the poor prognosis of this malignancy. ATC patients have a median survival of 3–6 months and a 20% 1-year survival rate [2–8].

Nearly all patients with ATC present with a cervical mass. However, regional or distant spread is apparent at the time of initial diagnosis in 90% of cases [9, 10] The lungs are the most common site of distant metastases (90%). Approximately 5–15% of patients have bone metastases and 5% brain metastases [10]. All patients are classified as TNM stage IV (A, B, or C) at presentation. T-stadium was formerly classified as T4, regardless of their size and overall tumor burden [2], but is staged like other thyroid cancers since 2016 (UICC 8th edition) [3, 11, 12].

Treatment of patients diagnosed with ATC is not well standardized and for many years the feasible options included surgery, radiotherapy, chemotherapy. The combination of these treatment modalities may maximize the clinical outcome, in terms of both local and systemic disease control, but doesn’t improve survival [11–14]. Surgery followed by chemoradiotherapy can significantly prolong the survival of patients carrying small, intrathyroidal tumors, but this kind of presentation is very unusual for this cancer [15]. Although up to 80% of the patients may initially respond to radiation, most have local recurrences [16]. Doxorubicin +/− Cisplatin is the most commonly used chemotherapy against ATC, but results have been disappointing [17–20]. Paclitaxel or docetaxel has shown some improvement in response in regionally confined ATC but not in regards to survival [21–23]. In patients with advanced disease, palliation of symptoms is of high priority [24]. Consequently, there is a critical need to develop novel systemic therapeutic approaches.

### Clinical trials of kinase inhibitors in anaplastic thyroid cancer

As ATC at the molecular level is highly pleiotropic with multiple mutations of oncogenes and tumor suppressor genes, a large number of proteins involved in critical cellular functions are over- or underexpressed [25]. Therefore targeted agents might represent a viable therapeutic option. Several targeted agents have been tried in ATC, with some evidence of activity (Tables 1, 2).

**Table 1:** Molecular targets of the kinase inhibitors and antibodies mentioned in text

TKI/Ab	RET	BRAF	RAF-1	mTOR	cMET	c-Kit	VEGFR1	VEGFR2	VEGFR3	PDGFRα	PDGFRβ	EGFR	MEK	BCR/ABL	NTRK
Axitinib							x	x	x						
Imatinib	x					x				x	x			x	
Sorafenib		x	x					x	x		x				
Sunitinib	x					x	x	x	x	x	x				
Pazopanib						x	x	x	x	x	x				
Vandetanib	x							x	x			x			
Erlotinib												x			
Gefitinib												x			
Dabrafenib		x^a^													
Vemurafenib		x^a^													
Cobimetinib													x		
Trametinib													x		
Everolimus				x											
Cetuximab												x			
Bevacizumab							x	x							
Lenvatinib	x					x	x	x	x	x					
Larotrectinib															x

*TKI* Tyrosine kinase inhibitor, *Ab* Antibody

^a^Mutant BRAF(V600E)

**Table 2:** Clinical studies with targeted therapies in patients with anaplastic thyroid cancer

Drug	Patients (n)	CR n (%)	PR n (%)	SD n (%)	Median PFS (months)	Median OS (months)	6mo-OS	Author (Reference)
**Imatinib**	8		2 (25%)	4 (50%)	6 (36%)		45%	Ha et al., JCO, 2010 [26] Phase II
**Sorafenib**	20		2 (10%)	5 (25%)	1.9	3.9	30%	Savvides et al., Thyroid, 2013 [27] Phase III
**Pazopanib**	15		0	0	2.0	3.6	13%	Bible et al., JCEM 2012 [28] Phase II
**Everolimus**	7		0	3 (43%)	2.2	2.5	0%	Lim et al., Ann Oncol., 2013 [29] Phase II
**Everolimus**	7		0	0		4.3		Schneider et al. J Clin Endocrinol Metab. 2017 [30] Phase II
**Everolimus**	7		1 (14%)	1 (14%)	2.2	4.6		Hanna et al. Clin Cancer Res. 2018 [31] Phase II
**Fosbretabulin** **(CA4P)**	3	1 (33%) CR > 30 mo		0				Dowlati et al., Cancer Res, 2002 [32] Phase II
**Fosbretabulin**	26		0	7 (27%)	3 (23%)	4.7	34%	Mooney et al., JCO 2006 [33] Phase II
**Fosbretabulin/carboplatin/paclitaxel**	80		20%	40%	3.3	5.2	26% (1y)	Sosa et al., Thyroid, 2014 [34] Phase III
**Axitinib**	2		1 (50%)					Cohen et al., JCO 2014 [35] Phase II
**Gefitinib**	1		1 (100%, > 4 mo)					Fury et al., Cancer Chemother Pharmacol 2007 [36] Phase I
**Gefitinib**	5		0	1 (20%, > 12 mo)				Pennell et al., Thyroid, 2008 [37] Phase II
**Larotrectinib**	7		2 (29%)	1 (14%)				Cabanillas et al., Ann Oncol 2020 [38] Phase II
**Sunitinib**	4		0	1 (25%)	9.8			Ravaud et al. Eur J Cancer 2017 [39] Phase II
**Lenvatinib + Pembrolizumab**	6	4 (66%)		1 (16%)	16.5	18.5	83%	Dierks et al. Thyroid 2021 [31] retrospective data
**Dabrafenib + Trametinib**	16	1 (6,2%)	10 (62%)	3 (19%)			62%	Subbiah et al. JCO 2018 [40] Phase II
**Pembrolizumab (1 pat Nivolumab)**	13			16% ORR	1.9	4.4	38% (1y)	Hatashima Thyroid 2022 [ 41] Case series
**Dabrafenib + Trametinib, Vemurafenib**	17, 4				6.4	14.5		De la Fouchardière Ann Endocrin 2022 [42] retrospective data

*CR* Complete response, *PR* Partial response, *SD* Stable disease, *PFS* Progression free survival, *OS* Overall survival, *ORR* Overall response rate, *mo* Months, *y* Year, *CA4P* combretastatin A4 phosphate

### Tyrosine kinase inhibitors other than vemurafenib

Imatinib can specifically inhibit c-Kit, Bcr-Abl tyrosine kinases and platelet-derived growth factor (PDGF) receptors, which are overexpressed in ATC. Ha et al. [26] treated 8 patients with advanced ATC with imatinib (400 mg *bid*); among them 2 obtained a partial response (120 and 694 days respectively) and 4 stable disease. The rate of 6-month progression-free survival (PFS) was 36% and the rate of 6-month overall survival was 45%.

Sorafenib is a tyrosine kinase inhibitor (TKI) of the Raf-1 protein kinase receptor, vascular endothelial growth factor receptor (VEGFR2,3) and PDGFβ and displays strong antiangiogenic activity. Savvides et al. [27] assessed the activity of sorafenib in a phase II study, in 20 patients with advanced ATC (400 mg *bid*). Two of the 20 patients had a partial response (10 and 27 months respectively), and 5 had stable disease. The 6-month PFS was 15% and the 6-month survival was 30%.

Pazopanib is a potent inhibitor of c-Kit, VEGFR1,2,3 and PDGFRα/β, fibroblast growth factor receptor 1 and 3 (FGFR1,3), and demonstrates evidence of in vivo antitumor activity in ATC [28] and enhances the cytotoxic effects of paclitaxel in vitro and in vivo in preclinical ATC models [43]. However, a phase II multicentre trial assessing pazopanib in ATC patients was quite disappointing revealing no responses. The treatment had to be discontinued due to disease progression or severe toxicity [28]. The median PFS was 62 days and the median survival 111 days. McLarnon et al. also showed that pazopanib alone is not effective against ATC [44].

Sunitinib is a multi-targeted inhibitor of VEGFR1,2,3, PDGFRα/β and RET. Preclinical studies showed little or no effect on the growth or differentiation of ATC cells [45]. However, Grande et al. reported an anecdotal experience of sunitinib in ATC, where the treatment induced an almost complete regression of the neck tumor mass despite having no impact on distant metastases [46]. The patient died as a result of a massive upper gastrointestinal bleeding 5 months after the start of sunitinib and while the patient was still on treatment [46]. A long-term survival with sunitinib (> 12 months) in an ATC patient has additionally been reported by Koussis et al. [47]. Finally, Schoenfeld et al. [48] reported on prolonged survival (> 18 months) and complete response in a patient with significant residual ATC in the neck following surgery, who was treated with chemoradiation with docetaxel along with concurrent and maintenance sunitinib. One clinical trial with sunitinib [39] reported a stable disease in one out of four patients with ATC.

Crizontinib is an anaplastic lymphoma kinase (ALK)-specific inhibitor. ALK is an oncogene, a member of the insulin receptor subfamily of receptor tyrosine kinases [49]. ALK-rearrangement has been recently described by Hamatani et al. [50] in 10 of 19 patients (atomic bomb survivors) with radiation exposed papillary thyroid carcinoma, with no detectable gene alterations in BRAF, RET, NTRK1, or RAS. A 71-year-old patient with ALK-rearranged ATC and no history of exposure to ionizing radiation demonstrated an excellent partial response (90%) to crizotinib (250 mg *bid*) for at least 6 months.

Everolimus is an allosteric inhibitor of the mammalian target of rapamycin (mTOR), the main kinase among the PI3K downstream effectors. It selectively suppresses the proliferation of ATC cells in preclinical setting [51]. In a phase II study in locally advanced or metastatic thyroid cancer of all histologic subtypes, everolimus achieved SD in five out of six patients, and marked tumor shrinkage in one of them. The latter patient had a near-complete response that lasted for 18 months. Whole-exome tumor sequencing in this patient revealed a somatic nonsense mutation (Q1178*) in the tumor-suppressor gene TSC2 that inactivates the gene, allowing for activation of the mTOR pathway [52]. Once resistance emerged in this patient, the mechanism of acquired resistance to everolimus was identified as a mutation in mTOR that prevented everolimus from binding to mTOR [52]. Inactivating mutations in the tumor-suppressor genes TSC1, TSC2, and STK11 are described as targets of TOR inhibitors in hamartoma syndromes [53] and in urothelial carcinoma [54].

Larotrectinib is a selective inhibitor of 3-tropomysin receptor kinase protein (TRK). It is approved by FDA and EMA for various cancers harboring a TRK fusion. 7 ATC patients with a NTRK fusion were treated, the overall response rate was 29% [38].

### Anti-angiogenic drugs

Fosbretabulin (formerly combretastatin A4 phosphate, CA4P) is a novel vascular-disrupting agent that targets existing tumor neovasculature, and causes an acute reduction in tumor blood flow and has antitumor activity against ATC cell lines [55]. One patient with ATC who received fosbretabulin monotherapy in a phase I study with advanced solid tumors experienced a durable complete response (> 30 months) however, the drug was found to be associated with significant cardiovascular side effects [32]. In a phase II study of fosbretabulin in 26 patients with ATC no patient experienced an objective response; the best response to treatment was stable disease in 7 patients, however median survival was about 5 months with 34 and 23% alive at 6 and 12 months, respectively [56]. In a study of fosbretabulin in combination with carboplatin/paclitaxel, one patient experienced a partial response and a second experienced stable disease for more than 4 months [57]. In a randomized study testing this combination therapy in 80 patients with ATC (carboplatin/paclitaxel (CP) versus CP/ fosbretabulin) median overall survival (OS) was 5.2 months for the CP/fosbretabulin arm and 4 months for the CP arm (*p* = 0.22). One-year survival for CP/fosbretabulin was 26% versus 9% for CP. There was no significant difference in PFS between the two arms [34].

Axitinib is a potent and selective inhibitor of VEGFRs (1, 2 and 3) [58, 59]. A phase II clinical trial was conducted in 60 patients with various types of advanced thyroid cancer (two with ATC), with an objective response in one of the two patients [35, 60]. Responses were noted in all histological subtypes.

### Anti-epidermal growth factor receptor (EGFR) drugs

EGFR is a transmembrane receptor tyrosine kinase which is overexpressed in ATC cell lines [61]. The activity of EGFR-inhibitors is tested mainly in preclinical trials [62]. Gefitinib is an EGFR inhibitor that blocks EGFR-mediated downstream signal transduction. In a phase I trial of Gefitinib in 18 patients with advanced solid tumors the only patient with ATC experienced a significant partial response for approximately 4 months before coming off study due to pulmonary embolism [36]. Because of the preclinical data, an open-label phase II study was initiated to examine the effectiveness of gefitinib in a mixed cohort of thyroid cancer patients [37]. Although there were no complete or partial responses in the 25 patients evaluated, one patient with ATC had stable disease beyond 12 months of therapy.

Erlotinib is an EGFR tyrosine kinase inhibitor with recently reported in vitro and in vivo antiproliferative effects in ATC [63, 64]. Several individual patients with partial response to erlotinib are reported [25, 65, 66]. A 65-year-old patient with EGFR-mutated ATC showed marked clinical response to erlotinib and remained progression free for more than 6 months [66].

Cetuximab is a human-murine chimeric monoclonal antibody against EGFR. In preclinical trials, Kim et al. [67] observed that combination therapy with cetuximab/irinotecan inhibits the growth and progression of orthotopic ATC xenografts in nude mice. A 61-year-old patient with EGFR-mutated ATC showed partial response to cetuximab in combination with intensity modulated radiation therapy. Her response lasted for 12 months; after local progression she received erlotinib and died of locoregional complications after 3 months [25].

## Case presentation

**Fig. 1:**
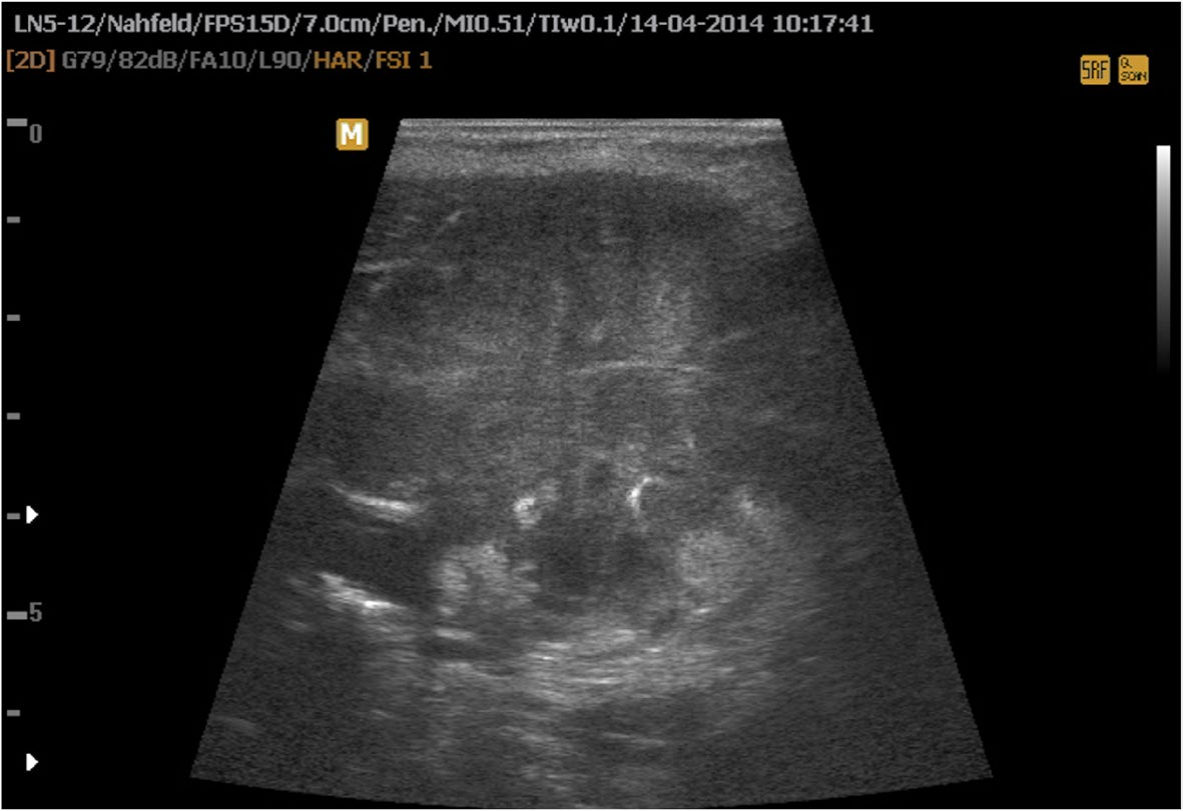
Ultrasound imaging: large left sided mass with inhomogeneous tissue, ill defined borders, and infiltration of muscle. For a video clip see supplementary file

**Fig. 2:**
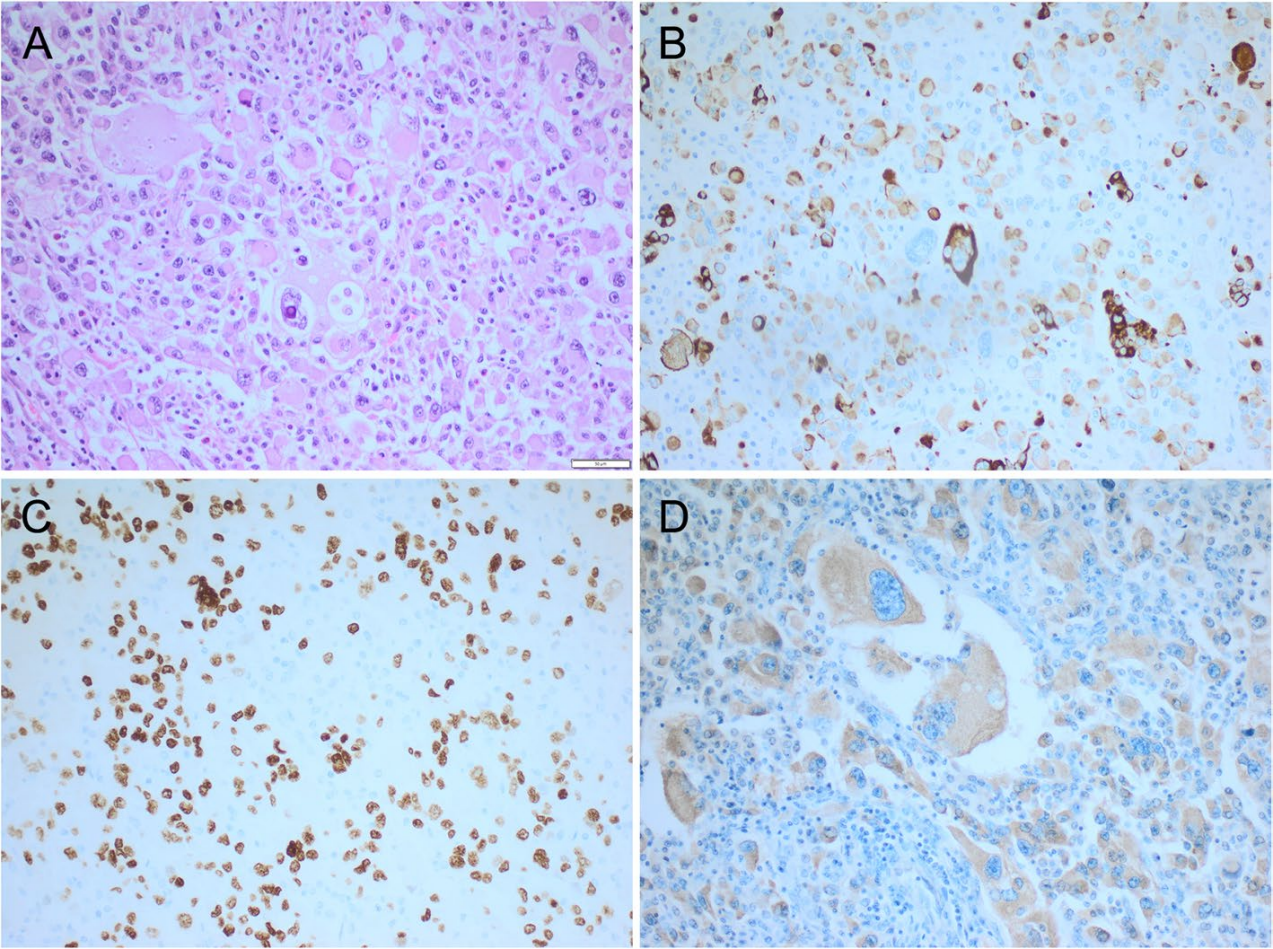
**A** Pleomorphic tumor cells with intermixed tumor giant cells with prominent nucleoli arranged in a loose pattern with admixed inflammatory cells. **B** The tumor cells reveal a variable expression of keratin 18. **C** TTF1 is detected in tumor cell nuclei. **D** Tumor cells are positive after incubation with a mutation specific BRAF^V600^ antibody. Note, negativity of admixed inflammatory cells. Original magnification each 200-fold

A 78-yr-old man presented in March 2014 with a 5-week history of an enlarged neck mass, neck pain and B symptoms. A mass at the left thyroid lobe and unilateral cervical lymphadenopathy were detected on physical examination and by cervical ultrasound. The fine needle aspiration biopsy showed a low differentiated pleomorphic carcinoma with a proliferation rate (Ki-67 index) of 75%. Neck ultrasound (US, Fig. 1), magnetic resonance imaging (MRI) and computed tomography (CT) confirmed the cervical mass on the left side with no clear demarcation from the thyroid gland and the sternocleidomastoid muscle, due to a large, partially hemorrhagic lymph node. The mass surrounded the left internal carotid artery and compressed the left internal jugular vein. Additionally disseminated intrapulmonary nodules were detected. For tumor debulking, thyroidectomy and resection of the left internal jugular vein were performed. Intraoperatively a locally advanced tumor with infiltration of the trachea was detected. In order to save the trachea, a R2 resection without further resection of lymph nodes was performed. Histology **(**Fig. 2**)** showed an anaplastic thyroid carcinoma (TNM: pT4b Nx M1; V1; stage IVc) and components of a papillary thyroid carcinoma with a positive BRAF-mutation (c.1799 T > A; p.V600E). Postoperatively no recurrent laryngeal nerve paralysis or hypoparathyroidism was detected. A postoperative CT-scan, 20 days after surgery, showed a rapid and marked progression of the pulmonary metastases as well as the locoregional, cervical, hilar and mediastinal lymph nodes.

**Fig. 3:**
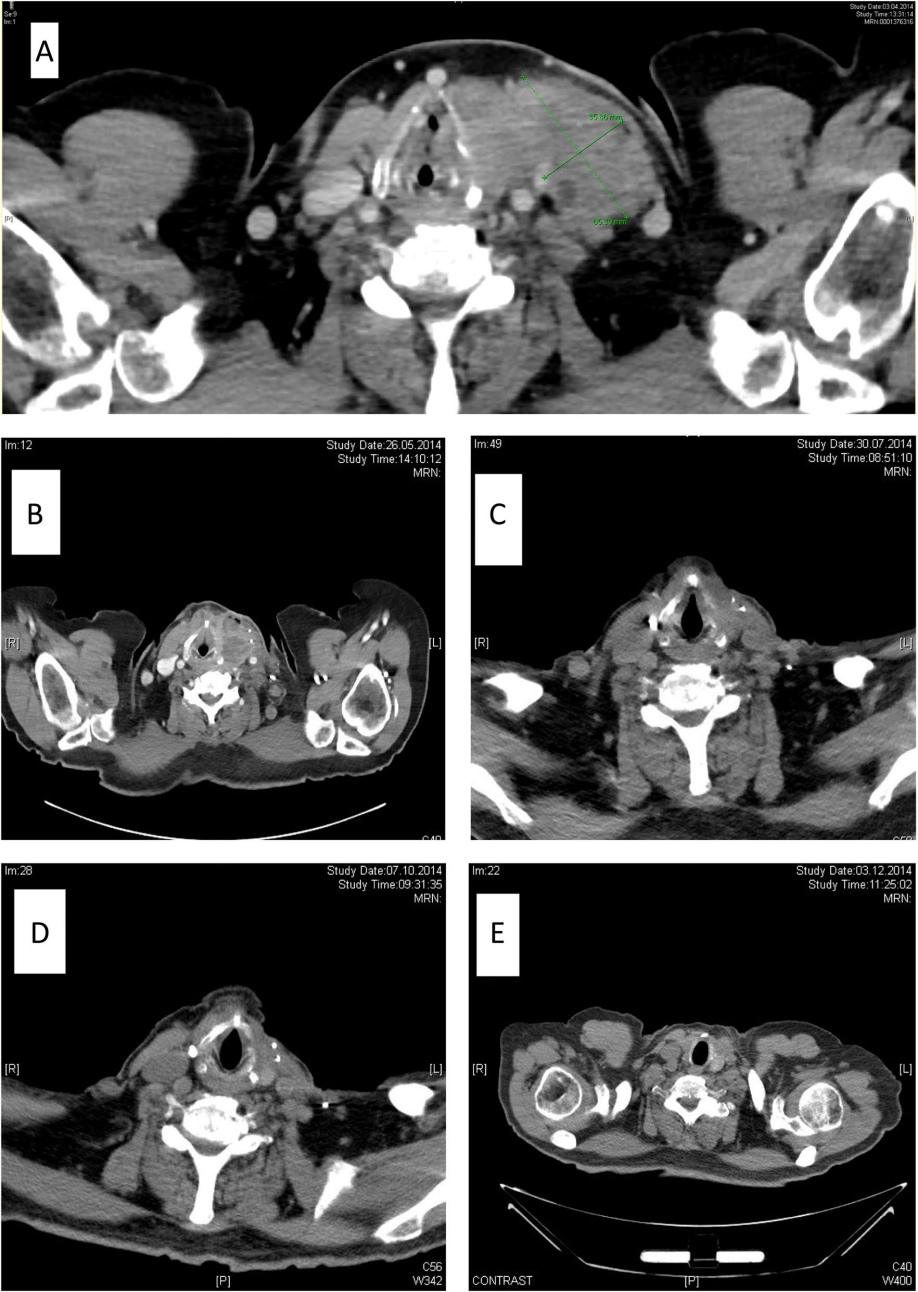
CT-scan, left sided thyroid tumor; **A** initial situation, tumor size 65 × 37 mm. **B** postoperative status on day 20; tumor size 44 × 26 mm. **C** status 2 months after initiation of vemurafenib; tumor size 35 × 22 mm. **D** status 5 months after initiation of vemurafenib; tumor size 33 × 16 mm. **E** status 7 months after initiation of vemurafenib; tumor size 33 × 16 mm

In May 2014, we initiated a first line adjuvant therapy with the oral BRAF inhibitor vemurafenib (960 mg *bid*). The condition of the patient improved rapidly, and he was discharged on day 7 after starting vemurafenib. A CT-scan on day 36 showed a significant partial remission of the cervical residual tumor, the lymphatic and pulmonary metastases (Fig. 3). Within 5 months, follow-up scans showed an almost complete regression of the pulmonary and lymph node metastases and consistent regression of the residual tumor in the neck. A single pulmonary nodule in segment 6 of the left lung showed a continuous progression (from initially 1.1 cm to 4.3 cm). During treatment the patient developed a xerosis cutis, an itchy eczema, and sporadic papulopustular lesions, treated with a consistent skin moisturizing regimen and antihistamines. Because of B symptoms and fatigue a sip feed nutrition with high energy density was initiated. The patient also developed an acute-on-chronic renal failure. In the overall constellation, we reduced the dose of vemurafenib (720 mg *bid*). After 7 months of sustained response to vemurafenib, scans revealed progressive disease with new cerebral metastases and new soft tissue metastasis in the right upper arm. The patient received radiotherapy and died in January 2015 6 days after the last radiation and 10 months after first diagnosis.

## Discussion and conclusion

TKIs are considered the most suitable systemic treatment for iodine-refractory differentiated thyroid cancer (DTC) [68] and advanced, progressive medullary thyroid cancer (MTC) [69]. The role of TKIs in ATC is an evolving field. Based on the results of a safety trial on lenvatinib this drug got approved by Japanese authorities for the treatment of ATC [70]. Consecutive trials reported disappointing results [71], one phase 2 trial was halted for futility as the minimum overall response rate threshold of 15% was not met upon interim analysis [72]. A recent German retrospective analysis [31] shows very promising data of 66% complete responses with the upfront combination of lenvatinib and pembrozilumab, an immune checkpoint inhibitor targeting programmed cell death protein 1 (PD-1). As ATC have a high PD-Ligand 1 (PD-L1) expression, a response could be expected. As the response time of checkpoint inhibitors alone is more than 8 weeks, its effect is limited by the rapid tumor growth of ATC. A phase II trial is initiated [73].

The effect of many targeted agents such as ubiquitin-proteasome inhibitors (bortezomib) and aurora kinase inhibitors (tozasertib, barasertib) has been evaluated in a series of ATC carcinoma lines. The major concern about these studies is that only few of them have been reproduced in vivo [74, 75]. Other drugs such as AEE788 or vandetanib (dual EGFR and VEGFR inhibitors) and angiogenic compounds such as bevacizumab (a monoclonal antibody against VEGF) have also been evaluated in preclinical in vitro and in vivo studies [76–78]. The dual MEK- and mTOR-inhibition (selumetinib, rapamycin) has also been tested in ATC lines and xenograft models [79]. The antitumor activity of these agents against ATC, eventually in combination with chemotherapeutic agents, makes them attractive candidates for further clinical development for the treatment of ATC.

To date, anecdotal experiences and small-sample clinical studies have been published on the effects of TKIs in ATC. Imatinib, Axitinib, Sorafenib, CA4 and other targeted agents have been tested in clinical trials, with encouraging activity. However, the median overall survival in these trials doesn’t exceed 6 months, and only 30–40% of the patients are alive after 6 months and 20% after a year [26, 27, 35, 56, 80]. Marked tumor shrinkage has been observed in a few case reports with targeted therapy after molecular tumor sequencing [52, 65, 66, 81].

BRAF is a serine- or threonine-specific protein kinase in the mitogen-activated protein kinase pathway, which regulates cell division and survival. The activating mutant protein BRAF (V600E) is detected in cutaneous melanoma, classic hairy-cell leukemia and papillary thyroid cancer [82, 83]. The BRAF inhibitor vemurafenib (PLX4032) improves survival and induces a response among patients with metastatic melanoma [84] and hairy-cell leukemia [85]. Cases of successful treatment have been also reported in progressive BRAF-mutated anaplastic pleomorphic xanthoastrocytoma and glioma in pediatric patients [86, 87].

Frequency of a BRAF (V600E) mutation in anaplastic thyroid carcinoma, which is thought to be derived mainly from papillary carcinoma by multi-step carcinogenesis, is about 25% - much lower than that in papillary carcinomas [83]. In a mouse model, the BRAF inhibitor PLX4720 suppressed growth of mutated human anaplastic thyroid cancer [88]. If downregulation of BRAF with BRAF inhibitors also causes sodium iodide symporter upregulation, as suggested by in vitro data, it could be expected that patients treated with BRAF inhibitors may undergo both reduction of tumor size/invasiveness and possible redifferentiation [88, 89]. A first case report published 2013 in NEJM [4], described a dramatic benefit of BRAF inhibition with vemurafenib in BRAF mutated ATC, while others observed a rapid progression after 2 months of therapy [90]. A phase II basket study including 7 ATC patients reports one complete response and one partial response, while in one patient follow up data were missing and four others had disease progression [91]. All patients had at least one prior systemic therapy and six prior radiation.

Our report describes the first complete case with marked response to first line adjuvant vemurafenib in ATC, followed by relapse after 7 months of therapy and a fatal outcome. Since resistance emerged in this patient, possibly combined targeted therapy respectively additional MEK inhibition may have improved the outcome,

McFadden et al. [92] have genetically engineered a mouse model of BRAF-mutant ATC and demonstrated that combination treatment with MEK (PD0325901) and BRAF inhibitors (PLX4720) results in enhanced antitumor activity as compared to treatment with a BRAF inhibitor alone, suggesting that this combination could be useful as a component of treatment regimens also in human. Combined BRAF and MEK inhibition has shown promising data in BRAF-mutated metastatic melanoma patients. Cobimetinib (GDC-0973, XL-518) and trametinib are MEK inhibitors. A phase III trial showed that cobimetinib in combination with vemurafenib increased the rate of complete/partial response as well as the progression-free survival in BRAF-mutated melanoma patients compared to vemurafenib alone [93]. An ongoing clinical trial aims to assess this combination in ATC, the primary completion date being estimated for July 2023 [94].

A combination of the BRAF inhibitor dabrafenib and trametinib improved the rate of progression-free survival in previously untreated patients who had metastatic melanoma [95] or ATC [40] with BRAF(V600E) mutations. Since May 2018 this combination has been FDA-approved in ATC. Nevertheless, the majority of patients face progressive disease even when treated with a combination of these agents. Recent french data suggest comparable efficacy of vemurafenib and dabrafenib plus trametinib, with a median overall survival of 14 months [42]. Mechanisms of resistance to BRAF inhibition have been extensively investigated, whilst less is known about the specific mechanisms of resistance to combined therapy [96].

The development and the utilization of multiple cancer-targeting agents are emerging strategies for ATC treatment. To date the BRAF pathway and checkpoint inhibition of PD1 are the two most promising therapeutic options. Vemurafenib has been the first drug to show a dramatic response in ATC patients. Our case report of an ATC patient is the first to show the postoperative benefit of vemurafenib in a first line setting, resulting in a marked improvement in quality of life. The effect was limited by acquired resistance to targeted therapy. Combined BRAF and MEK inhibition may give more beneficial results, although recent data on ATC do not support this assumption. Further molecular characterization and investigation of mechanisms of resistance is mandatory in this field. Ongoing clinical trials will further define the role of TKIs, checkpoint inhibitors, and the impact of BRAF and MEK inhibition on ATC.

## Supplementary Information

**Additional file 1.**

## Data Availability

The datasets used and/or analyzed during the current study are available from the corresponding author on reasonable request.

## Abbreviations

AJCC: American Joint Committee on Cancer
ALK: Anaplastic lymphoma kinase
ATC: Anaplastic thyroid cancer
CA4P: Combretastatin A4 phosphate
CT: Computed tomography
CP: Carboplatin/paclitaxel
DTC: Differentiated thyroid cancer
EGFR: Epidermal growth factor receptor
FGFR: Fibroblast growth factor receptor
MRI: Magnetic resonance imaging
MTC: Medullary thyroid cancer
NTKR: Neurotrophic tyrosine kinase receptor
OS: Overall survival
PDGF: Platelet-derived growth factor (PDGF)
PFS: Progression free survival
PR: Partial response
SD: Stable disease
TKI(s): Tyrosine kinase inhibitor(s)
TRK: Tropomyosin receptor kinase
US: Ultrasound imaging
VEGFR: Vascular endothelial growth factor receptor
